# Supplementary material for: Revisiting the Dissolution of Praziquantel in Biorelevant Media and the Impact of Digestion of Milk on Drug Dissolution
Source: Pharmaceutics. 2022 Oct 19;14(10):2228. doi: 10.3390/pharmaceutics14102228 (PMC9609124; doi:10.3390/pharmaceutics14102228)
Supplement: Supplementary file 1 [file pharmaceutics-14-02228-s001.zip › pharmaceutics-1901181-supplementary.pdf]

*Supplementary Materials*

# Revisiting the Dissolution of Praziquantel in Biorelevant Media and the Impact of Digestion of Milk on Drug Dissolution

**Thomas Eason [1], Gisela Ramirez [1], Andrew J. Clulow [1,2], Malinda Salim [1] and Ben J. Boyd [1,3,*]**

[1] Drug Delivery Disposition and Dynamics, Monash Institute of Pharmaceutical Sciences, Monash University (Parkville Campus), 381 Royal Parade, Parkville, VIC 3052, Australia
[2] BioSAXS Beamline, Australian Synchrotron, ANSTO, 800 Blackburn Road, Clayton, VIC 3168, Australia
[3] Department of Pharmacy, University of Copenhagen, Universitetsparken 2, 2100 Copenhagen, Denmark
* Correspondence: ben.boyd@monash.edu; Tel.: +61-3-99039112; Fax: +61-3-99039583

**Table S1**. Nutritional information of milk and infant formula (IF; based on reconstitution of 14.0 g powder) per 100 mL.

| Nutritional Information | Quantity per 100 mL | |
|---|---|---|
| | Milk | IF |
| Total fat | 3.8 g | 3.8 g |
| Saturated fat | 2.5 g | 2.1 g |
| Total protein | 3.4 g | 1.5 g |
| Carbohydrate | 4.8 g | 7.9 g |
| Sodium | 40 mg | 36 mg |
| Calcium | 115 mg | 60 mg |
| Vitamin A | 41 μg | 60 μg |
| Riboflavin (vitamin B2) | 200 μg | 112 μg |

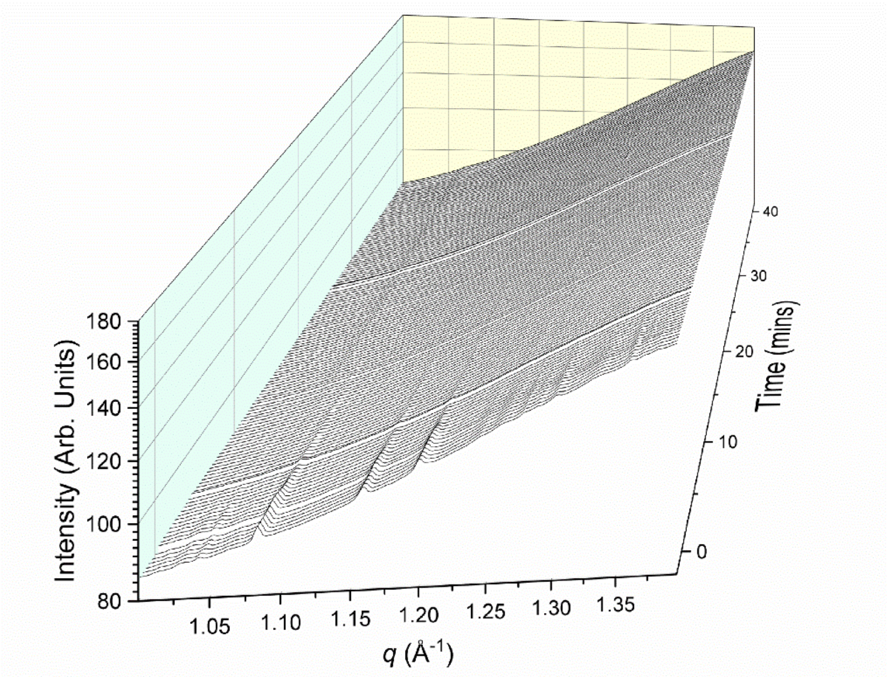


**Figure S1**. X-ray scattering pattern of praziquantel in 3.8% infant formula during dissolution (time < 0 min) and digestion (time > 0 min) showing the progressive decrease in peak intensity that is synchronous across peaks at different positions.
